# Spatial transcriptomics tools allow for regional exploration of heterogeneous muscle pathology in the pre-clinical rabbit model of rotator cuff tear

**DOI:** 10.1186/s13018-022-03326-8

**Published:** 2022-10-04

**Authors:** Severin Ruoss, Mary C. Esparza, Laura S. Vasquez-Bolanos, Chanond A. Nasamran, Kathleen M. Fisch, Adam J. Engler, Samuel R. Ward

**Affiliations:** 1 Department of Orthopaedic Surgery, UC San Diego, 9500 Gilman Drive, La Jolla, CA 92093-0863 USA; 2 Department of Bioengineering, UC San Diego, La Jolla, CA USA; 3 Center for Computational Biology and Bioinformatics, UC San Diego, La Jolla, CA USA; 4 Department of Obstetrics, Gynecology and Reproductive Sciences, UC San Diego, La Jolla, CA USA; 5 Department of Radiology, UC San Diego, La Jolla, CA USA

**Keywords:** Spatial transcriptomics, Visium, RNA-sequencing, Rotator cuff

## Abstract

**Background:**

Conditions affecting skeletal muscle, such as chronic rotator cuff tears, low back pain, dystrophies, and many others, often share changes in muscle phenotype: intramuscular adipose and fibrotic tissue increase while contractile tissue is lost. The underlying changes in cell populations and cell ratios observed with these phenotypic changes complicate the interpretation of tissue-level transcriptional data. Novel single-cell transcriptomics has limited capacity to address this problem because muscle fibers are too long to be engulfed in single-cell droplets and single nuclei transcriptomics are complicated by muscle fibers’ multinucleation. Therefore, the goal of this project was to evaluate the potential and challenges of a spatial transcriptomics technology to add dimensionality to transcriptional data in an attempt to better understand regional cellular activity in heterogeneous skeletal muscle tissue.

**Methods:**

The 3′ Visium spatial transcriptomics technology was applied to muscle tissue of a rabbit model of rotator cuff tear. Healthy control and tissue collected at 2 and 16 weeks after tenotomy was utilized and freshly snap frozen tissue was compared with tissue stored for over 6 years to evaluate whether this technology is retrospectively useful in previously acquired tissues. Transcriptional information was overlayed with standard hematoxylin and eosin (H&E) stains of the exact same histological sections.

**Results:**

Sequencing saturation and number of genes detected was not affected by sample storage duration. Unbiased clustering matched the underlying tissue type-based on H&E assessment. Connective-tissue-rich areas presented with lower unique molecular identifier counts are compared with muscle fibers even though tissue permeabilization was standardized across the section. A qualitative analysis of resulting datasets revealed heterogeneous fiber degeneration–regeneration after tenotomy based on (neonatal) myosin heavy chain 8 detection and associated differentially expressed gene analysis.

**Conclusions:**

This protocol can be used in skeletal muscle to explore spatial transcriptional patterns and confidently relate them to the underlying histology, even for tissues that have been stored for up to 6 years. Using this protocol, there is potential for novel transcriptional pathway discovery in longitudinal studies since the transcriptional information is unbiased by muscle composition and cell type changes.

## Introduction

RNA sequencing has been extensively used to define skeletal muscle tissue states, to track disease progression, phenotypic changes, and healing responses after treatment, and to investigate the mechanisms-of-action for respective treatments in clinical settings [1, 2] and pre-clinical models [3, 4]. Conditions affecting skeletal muscle, such as chronic rotator cuff tears, low back pain, whiplash, dystrophies, Huntington’s disease, and many others often share a similar change in muscle phenotype: contractile tissue portions decrease while adipose and fibrotic tissues increase [5–9]. Chronic rotator cuff tears affect approximately 50% of the population over 50 years of age, roughly a third of these tears are symptomatic, and most asymptomatic tears will become symptomatic within 3 years of diagnosis [10–13]. In these patients, outcomes after surgical repair are directly related to the grade by which contractile tissue has been replaced by adipose and fibrotic tissue, and this degenerative process continues even after repair [14–16]. To understand the pathobiology underlying this irreversibility of chronic rotator cuff tears and to find ways to mitigate contractile tissue loss, intramuscular fat accumulation, and fibrosis, scientists have been collecting biopsies intraoperatively at the time of surgical repair. Naturally, these different tissue types are defined by different cell types, thus the RNA extracted from muscle biopsies is a function of the relative portions of contractile, adipose, and fibrotic tissues present within the biopsy [1]. Until recently, this has been problematic because changes in cell types and numbers have limited and confounded our ability to understand the bulk transcriptional changes in cell states and mechanisms-of-action. With the advent of single cell RNA sequencing (scRNA-seq), investigators can now track changes in cell numbers and types, and precisely attribute transcriptional states to single cells. Muscle cells, however, can be several centimeters long [17] and thus, clearly exceed the length capacity of high-throughput droplet-based single-cell technologies. As an innovative alternative, single-nuclei RNA sequencing (snRNA-seq) has been employed for skeletal muscle [18–20]. This approach, too, has at least three noteworthy limitations for skeletal muscle: (1) muscle fibers are multinucleated cells—it is often unknown how many and which nuclei were derived from which cell, (2) the vast majority of RNA molecules are located outside the nucleus [21, 22] and therefore missed by snRNA-seq, and (3) there is significant heterogeneity in the spatial distribution of RNA molecules within muscle fibers [21, 22] and nucleus translocation to the fiber center during degeneration and regeneration [6, 7, 23]. None of these issues are resolved by snRNA-seq.

More recently, spatial transcriptomics have emerged and shown some promise to addressing these problems by adding a layer of spatial resolution [24, 25]. 10 × Genomics is one of the most common sequencing platforms, and we chose to use the Visium technology for this study. However, there are many different useful platforms available, each presenting with opportunities and limitations [26]. One of the advantages of the Visium technology is that it combines transcriptomics with standard hematoxylin & eosin (H&E)-based microscopy. H&E is a gold-standard qualitative tool used by clinicians and researchers to assess muscle fiber integrity, cross-sectional area, central nucleation, and to estimate muscle degeneration, fatty infiltration, and fibrosis [6, 7, 27, 28]. Unfortunately, skeletal muscle is typically not a prioritized tissue for companies to test and establish protocols which involve enzymatic permeabilization times and subsequent RNA quality and capture efficiency assessments. Therefore, we sought to establish a 10 × Visium muscle protocol in a previously validated pre-clinical model and to generate a pilot dataset that demonstrates the use and potential of this technology to investigate conditions affecting skeletal muscle.

Here, we were using the Visium 3′ spatial RNA sequencing technology by 10 × Genomics to identify regional differences in degenerating supraspinatus muscle tissue from the pre-clinical rabbit model of rotator cuff tear. Given that the cytosol of muscle cells is exposed by cryosectioning, we hypothesized that transcript capture efficiency is higher in these cells compared with mononuclear cells residing between fibers, despite homogenous permeabilization across the entire tissue section. Importantly, we were using tissues stored for over 6 years and compared results and quality to a freshly frozen sample with the goal to evaluate whether new tissue sources/animal experiments are needed or whether this technology can retrospectively be used in previously acquired tissues.

## Methods

### Tissue collection

**Fig. 1:**
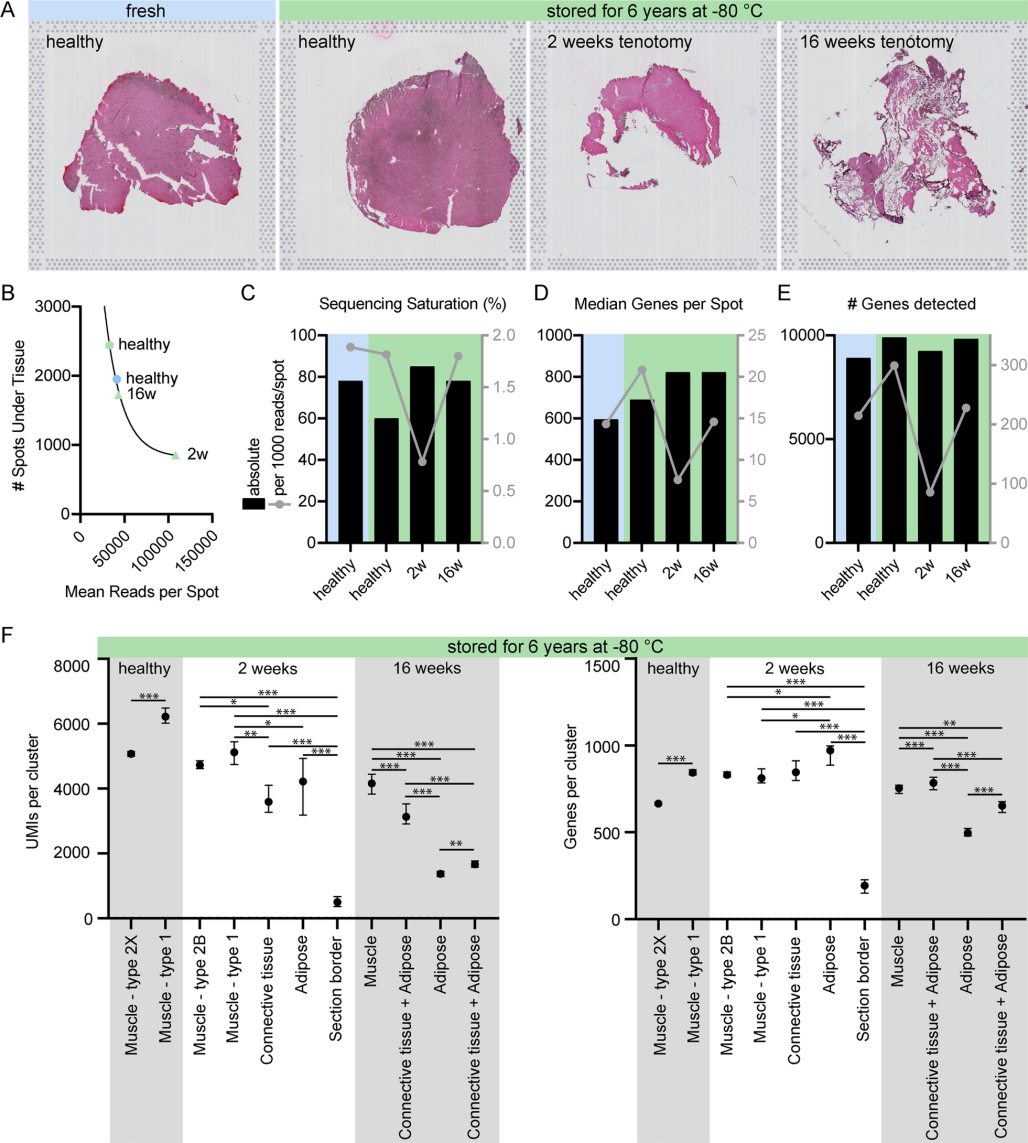
Baseline sequencing results comparison of fresh (blue) vs. stored (green) rabbit rotator cuff samples at different tear states. **A** H&E-stained histological sections on the Visium Spatial transcriptomics slide. **B** Increasing number of spots under tissue decrease mean reads per spot at a given total number of reads. **C** Absolute sequencing saturation (black, left Y-axis) and relative %-point of saturation added per 1000 reads/spot (gray, right Y-axis), **D** median genes per spot (black, left Y-axis) and relative median genes added per spot per 1000 reads/spot (gray, right Y-axis) and, **E** total number of genes detected (black, left Y-axis) and relative number detected per 1000 reads/spot (gray, right Y-axis) suggest that data quality was not affected by 6 years of sample storage. **F** Unique Molecular Identifier (UMI) and gene counts per sample relative to different clusters/underlying tissue types. The fresh sample was very homogenous and only yielded one high-quality tissue cluster vs. low-quality section border clusters, thus it was excluded from this piece of analysis. An example of such a low-quality section border cluster was included in the 2 weeks sample. Clusters were named based on the underlying tissue type identified using H&E. Different fiber type clusters were named based on differentially expressed MYH isoforms. Data in (**F**) are medians and 95% confidence intervals. *p < 0.05; **p < 0.01; ***p < 0.001

To replicate a rotator cuff tear, tenotomy of the supraspinatus was performed in the rabbit model, and tissues were harvested as previously described [3, 28, 29]. All animals were female New Zealand White and skeletally mature (6 months old, 4.35 ± 0.2 kg). The following four sections from the posterior distal region of the supraspinatus muscle belly, free from fascia and tendon/junction were used: one fresh healthy supraspinatus, one healthy supraspinatus stored for 6 years, one from 2-week post-tenotomy stored for 6 years, and one from 16-week post-tenotomy stored for 6 years (Fig. 1A). These time points were chosen purposefully to compare RNA quality as well as the feasibility of using tissue stored for approximately 6 years to a recently harvested tissue. All harvested samples were pinned to in vivo length and flash frozen in liquid nitrogen-chilled isopentane for storage at − 80 °C. Fractions of these muscles were then embedded in optimal cutting compound (OCT) media, cut into 10-µm sections using a cryostat, and mounted on 10 × Visium slides.

### Library preparation and bioinformatics

The 10 × Genomics Spatial RNAseq Visium platform was used for spatial transcriptomics experiments and experiments were performed according to the User guide (10 × Genomics, CG000239 Rev E). In summary, a 10 × Genomics Visium Gene Expression slide has four capture areas, each with an array of 5000 circular spots containing printed DNA oligos for mRNA capture. These oligos on each spot have a PCR handle, unique spatial barcode, unique molecular identifier (UMI), and a poly-dT-VN tail for capturing the 3′ end of mRNA molecules. Each spot with a unique spatial barcode is 55 µm in diameter and the center-to-center distance between spots is 100 µm. One 55-µm spot captures mRNA from approximately 10 to 20 cells depending on cell size and packing density which is variable across the tissue. SSP sections were then fixed in pre-chilled methanol for 30 min and then H&E stained and imaged. The optimal permeabilization time for 10-µm-thick rabbit skeletal muscle sections was found to be 18 min using the 10 × Genomics Visium Tissue Optimization Kit. Spatially tagged cDNA libraries were created using the 10 × Genomics Visium Spatial Gene Expression 3′ Library Construction V1 Kit. H&E-stained skeletal muscle tissue sections were imaged using a slide scanner system. Second strand synthesis was performed, followed by denaturation and transfer of the cDNA from the slide to a PCR tube. qPCR (Biorad CFX384) was used to determine the number of cDNA amplification cycles required. After cDNA amplification, a cleanup was performed, followed by a fragment analysis of the cDNA which was performed on a HS D1000 TapeStation (Agilent Technologies). Library construction followed with post-fragmentation end repair, A-tailing double side size selection, adaptor ligation, and cleanup. Sample index PCR was performed based on cDNA input followed by double sided size selection. Post-library construction QC was done using the TapeStation to determine average fragment size [30, 31]. Finally, samples were pooled for sequencing on a Novaseq 6000 (Illumina). Images were manually aligned using Loupe Browser 5.0 (10X Genomics). The Oryctolagus cuniculus reference genome was assembled using the mkref function [32] from Space Ranger 1.2.0 (10X Genomics) with OryCun2.0 (Oryctolagus_cuniculus.OryCun2.0.dna.toplevel.fa) and annotations from Ensembl release 104 (Oryctolagus_cuniculus.OryCun2.0.104.chr.gtf). Genes were quantified using the count function from Space Ranger 1.2.0 [33] and then explored in Loupe Browser v.6.0.0 (10 × Genomics). The number of clusters was determined by unbiased k-means clustering. Myofibers and nuclei were manually counted using ImageJ. Either all or twenty randomly chosen spots per cluster were counted, whichever came first. Spots at section borders were not included. UMIs, genes, myofiber, and nuclei numbers per cluster and per sample were compared in SPSS (v.26; IBM) using a univariate ANOVA with Fisher’s least significant difference test. The datasets are publicly available on Gene Expression Omnibus (GEO GSE210773).

## Results

The four H&E-stained samples are shown in Fig. 1A. Due to size differences between samples, and the fact that the number of total reads per section scales with the number of covered RNA capture spots, different sequencing depths (Mean Reads per Spot) were observed as expected (Fig. 1B). Accordingly, data were normalized to reads per spot to allow for comparisons between samples. While absolute sequencing saturation was the highest in the 2w sample, relative sequencing saturation was decreased in this sample (Fig. 1C). Absolute median genes per spot and total number of genes detected were higher in the 6-year stored (green) compared with the fresh sample (blue), but relative median genes/spot and relative total gene numbers were the lowest in the 2w sample (Fig. 1D + E) because absolute sequencing depth (Fig. 1B) and saturation (Fig. 1C) were higher than in the other samples. Importantly, there were significant differences in UMI and gene counts within samples, which was dependent on the underlying tissue type (Fig. 1F). Generally, more transcripts (UMIs) were detected in muscle-rich capture spots compared to connective and adipose tissue-rich spots. Conversely, the number of different genes detected was unaffected by UMI capture in connective tissue and was even higher in adipose-rich spots in the 2w sample (Fig. 1F). The fresh sample was not included in this analysis because it was very homogenous and yielded only one high-quality, muscle-based tissue cluster (data not shown). All other clusters of this sample were low-quality section border clusters. An example of such a low-quality section border cluster is shown in the 2-week sample (Fig. 1F). Interestingly, the muscle-rich spots subclustered into fiber types in the healthy and 2-week samples based on myosin heavy (MYH) and light chain (MYL) transcript expression (Fig. 1F).

**Fig. 2:**
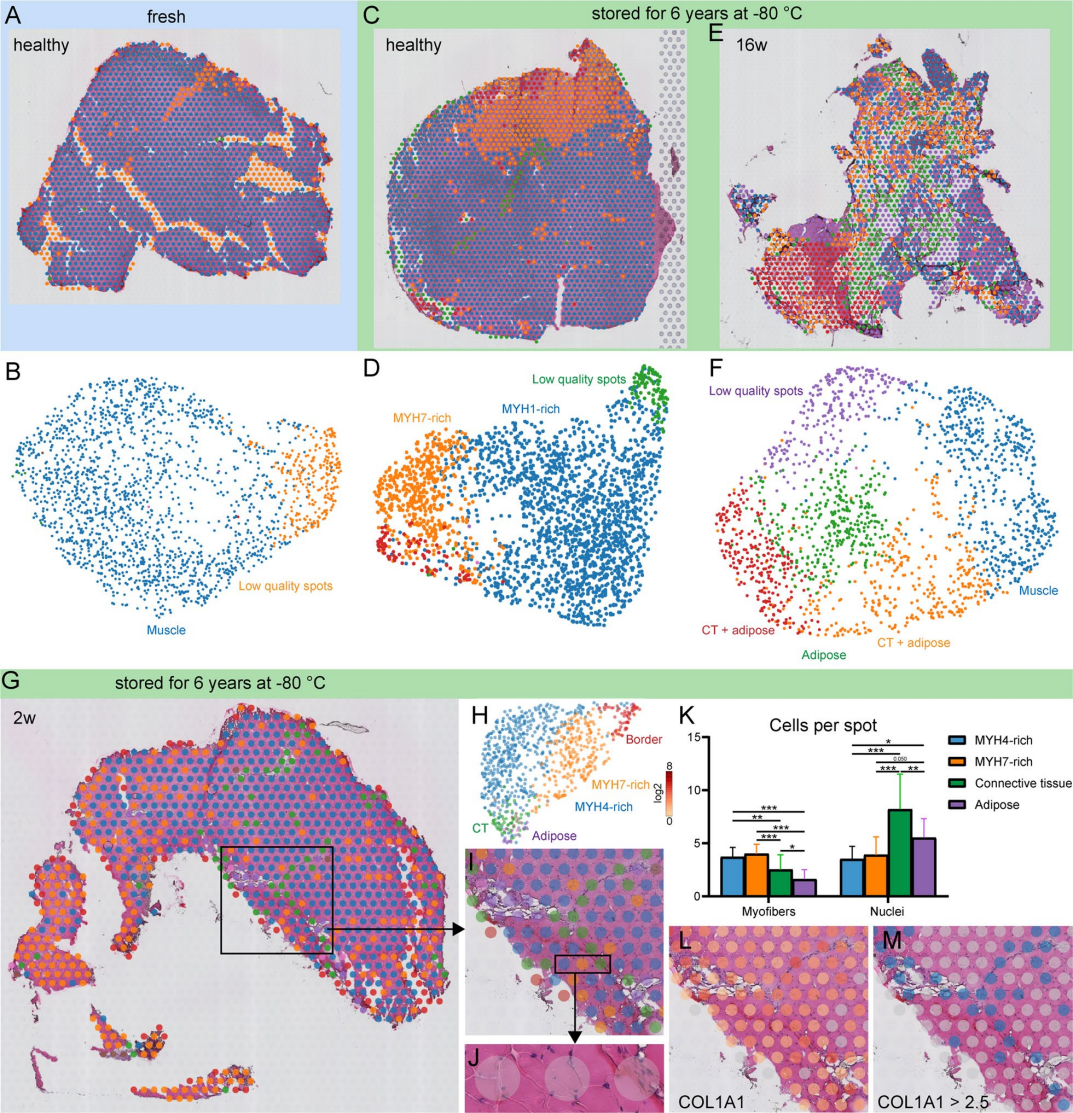
Cluster analysis suggests that transcriptional signatures correspond with the underlying tissue but are a conglomerate of cells and cell types per spot. **A**–**H** Unbiased k-means clustering spatially allocated to their original capture area and corresponding unbiased uniform manifold approximation and projection (UMAP) clustering of the exact same clusters. CT, connective tissue. **I** Illustration of how unbiased clustering corresponded to the underlying histology. The green, pink, brown, and purple clusters appeared to be derived from connective tissue- and adipose-rich areas, respectively, while orange and blue clusters represented muscle fiber-rich areas. **J** Transcripts were typically derived from multiple cells (and cell types) per spot: **K** Cluster-specific myofiber and nuclei numbers per capture spot differed depending on the underlying tissue type. **L** As a practical example of manual tissue identification by transcriptional markers, the connective tissue marker COL1A1 was detected in most capture areas, but **M** connective tissue-rich areas could be highlighted by setting a marker expression threshold. Data in (**K**) are means and standard deviation. *p < 0.05; **p < 0.01; ***p < 0.001

Unbiased k-means clustering corresponded to the underlying tissue types based on standard H&E histology (Fig. 2A, C, E, G, I). Unbiased uniform manifold approximation and projection (UMAP) plots, independent of spatial histological information, are displayed in Fig. 2B, D, F, H. Clusters were associated most often with dominant myosin heavy chain types, with connective tissue (green), or with adipose tissue (purple). A small number of clusters, typically located at section borders, sometimes with only partial tissue coverage, had lower quality RNA capture making association challenging (Fig. 2A–G). Importantly, as opposed to single-cell RNA sequencing, the transcripts per spot were typically derived from multiple cells (Fig. 2J), thus the spatial transcriptomics clustering information gained was a conglomerate of transcriptomes (Fig. 2K). Accordingly, it was more challenging to use classic transcriptional markers to find specific cells or tissues. For example, the connective tissue marker COL1A1 was detected in almost every capture spot (Fig. 2L). Transcript count thresholds had to be set in order to correlate the transcriptional marker with the underlying histology, and to use single genes as tissue- or cell-specific markers instead of unbiased clustering (Fig. 2M).

**Fig. 3:**
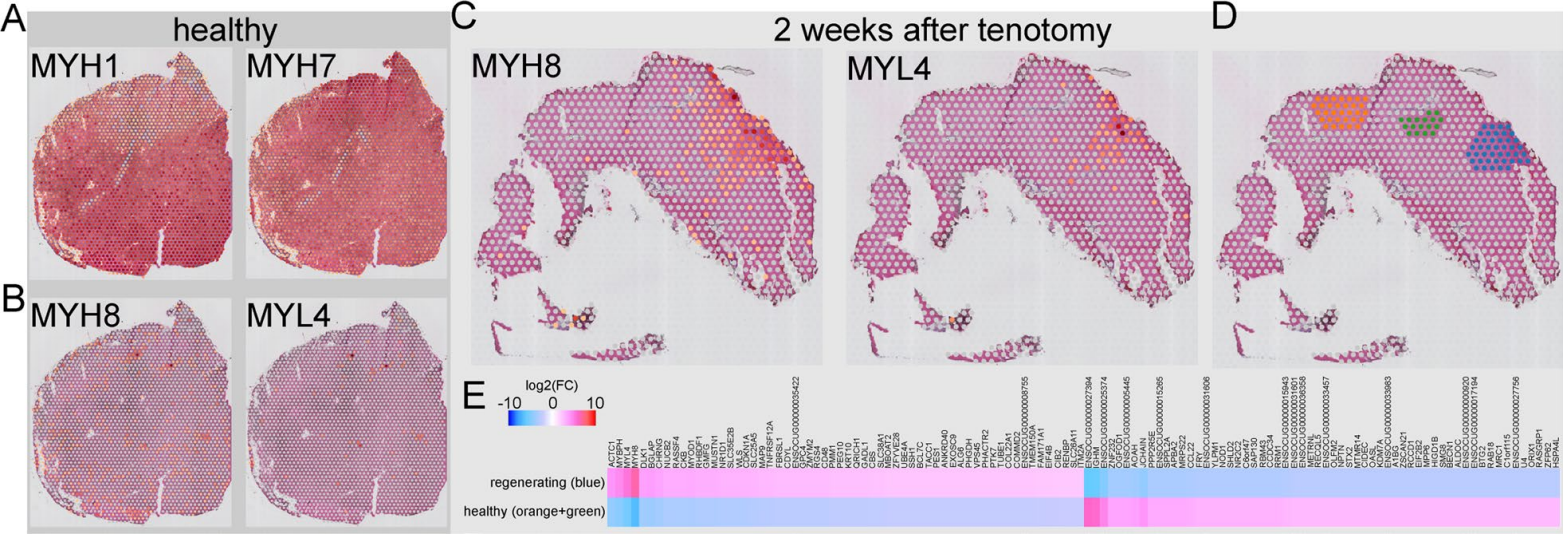
Potential applications of spatial transcriptomics. **A** Regional differences between fast type IIx (MYH1) and slow type I (MYH7) fiber portions in healthy rotator cuff. **B** Only a few very specific spots showed fiber degeneration/regeneration in healthy rotator cuff as indicated by neonatal myosin (MYH8) and myosin light chain 4 (MYL4) expression. Conversely, in (**C**) Muscle at 2 weeks after tenotomy presented with an entire area undergoing a degeneration/regeneration cycle. **D** Manual selection of the regenerating area (blue) based on MYH8 and MYL4 transcript detection and two randomly selected areas (orange and green) which i) did not present with those transcripts and ii) looked healthy based on H&E. These were then used for (**E**) Differentially expressed gene analysis of those healthy vs. regenerating muscle fibers. MYH8 and MYL4 were biased differentially expressed because they were used as a selection criterion for these areas, but the other differentially expressed genes may give us further transcriptional insights into regeneration or lack thereof in this pre-clinical setting. ENSOCUG sequences could not be annotated because we did not find corresponding gene IDs

To demonstrate the potential of this technology in skeletal muscle, we performed a proof-of-concept analysis specific to skeletal muscle, *i.e.*, fiber typing based on myosin heavy chain isotype (Fig. 3A), followed by an exploratory approach to identify regenerating muscle fibers and their transcriptional differences compared with their non-regenerating counterparts that appear to be healthy based on H&E (Fig. 3B–E). In healthy muscle, the predominantly detected myosin heavy chain transcripts were type 2x (MYH1) and type 1 (MYH7) fibers (Fig. 3A) followed by a few type 2b fibers (data not shown). MYH2 (type 2a) was not detected. The expression levels of MYH1 and MYH7 largely mirrored one another, as expected from different fiber types (Fig. 3A). Transcriptional expression of neonatal myosin heavy chain (MYH8) indicating fiber regeneration was sporadically detected (i.e., punctate) in a few fibers and colocalized with myosin light chain 4, another marker for regenerating fibers (Fig. 3B) [34]. Conversely, these transcriptional markers revealed an entire region of muscle undergoing degeneration/regeneration at 2 weeks after rotator cuff tear (Fig. 3C). As this cluster was not identified by unbiased clustering (Fig. 2A), we manually defined this area as “regenerating” based on MYH8 and MYL4 transcript detection and randomly chose two other areas that were; (1) areas histologically identified as healthy muscle based on standard H&E, and (2) areas not identified as regenerating by MYH8 and MYL4 transcript detection (Fig. 3D). Differential gene expression analysis of these regions revealed transcriptional differences between healthy and regenerating fibers that would otherwise look similar on H&E (Fig. 3E). In summary, this discovery demonstrated the huge potential of this technology to discover and explore new mechanisms and pathways. Key challenges and tips to approach them are summarized in Table 1. The datasets are available on GEO (GSE210773).

**Table 1:** Summary of key challenges, tips, and examples from current data

Step	Challenge	Tip	Examples from current data
Establishing permeabilization time	As a bright signal is desired, scientists may choose exposure times that are too long, too much gain, and/or laser strengths that are too high in an effort to obtain "better" images	Verify that there is no signal in the negative control and adjust parameters accordingly	
Choosing tissue section and size	Section has to fit capture area	Trim sample size in the cryostat using a pre-cooled razor blade	
	Mounting sections onto Visium slide is a final step	Before mounting section onto Visium slide, mount test sections onto standard microscopic slide to verify there is no freezing damage and the muscle fibers are oriented correctly	
	The same Cq value (as determined by qPCR) are used per slide for cDNA amplification, but optimal Cq values may differ between samples	Mount approximately similar section sizes and sample types per run	
	If the libraries are going to be pooled for sequencing and these libraries contain information from unequal numbers of capture areas, then sequencing reads are asymmetrically shared, leading to different sequencing depths	Mount approximately similar section sizes and sample types per run	The 2-week sample was too small to be pooled with the other samples (Fig. 1A), leading to unequal sequencing depths (mean reads per spot, Fig. 1B)
Choosing sequencing depth	How deep do these samples have to be sequenced? What is the relationship between false negative rate and sequencing depth?	As a general rule, increasing sequencing depth will increase sequencing saturation which decreases false negative rate. Based on the current data, this has to be addressed separately for each sample depending on tissue size and heterogeneity	Higher mean reads per spot leads to higher absolute sequencing saturation (Fig. 1A + B). Despite this, most genes were detected in the largest sample (Fig. 1E), even though sequencing depth and saturation was lowest in this sample (Fig. 1B + C)
Interpreting false negative rate per cluster or tissue type, respectively	The transcripts per spot are derived from more than one cell (Fig. 2D + E). The more heterogeneous these cells are, the more different transcripts are expected to be; thus, the deeper it must be sequenced	Increase sequencing depth depending on spots and tissue type of interest	More nuclei contribute to capture areas of connective tissue (Fig. 2E). Even though these spots tended to contain fewer RNA molecules compared with the cytosol of myofibers (Fig. 1B), the number of different genes is less affected, suggesting more heterogeneity (Fig. 1B)
Identification of areas of interest and investigating heterogeneity	The heterogeneity of cells contributing to a spot may exceed more subtle tissue-specific transcriptional states in unbiased clustering	Manually perform hypothesis-driven transcriptional marker search	Myofibers undergoing degeneration/regeneration cycles did not cluster separately (Compare Fig. 2A with Fig. 3C). Manual selection and differential gene expression analysis was performed using expression of key transcriptional markers (Fig. 3D + E)

## Discussion

The goal of this project was to establish the Visium spatial transcriptomics technology in a clinically relevant animal model for skeletal muscle diseases. The current data demonstrated the feasibility and potential of this technology to investigate skeletal muscle conditions. Importantly, valuable data were derived even from tissues that have been stored at − 80 °C for over 6 years.

The hypothesis that UMIs and number of different genes per cluster differed depending on the underlying tissue was confirmed. This is important to note because the Space Ranger Web Summary by 10 × Genomics reports these numbers only across all clusters [35] but decreased UMI and gene counts in certain clusters may limit the interpretation of the data because, accordingly, sequencing saturation may be locally overestimated in those clusters [35]. We hypothesize that UMI counts in spots predominated by muscle cells were higher because cryosectioning per se perpendicularly cuts through the muscle fiber, thus naturally exposing the cytosol and residing transcripts to the capture area of the slide, which would facilitate the activity of the permeabilization enzyme. Conversely, membranes of mononuclear cells residing in connective tissue between fibers may have remained intact after cryosectioning, leading to locally different efficiencies in the incubation with permeabilization enzyme. Interestingly, the number of different genes in connective tissue-rich capture spots appeared to be less affected by lower UMI counts. It is possible that higher heterogeneity of cell types in connective tissue-rich spots increased the number of different genes in these spots despite relatively lower UMI counts, because the connective tissue is vascularized and, thus, is not only home to fibroadipogenic progenitors and resident macrophages, but also endothelial cells, pericytes, smooth muscle cells, and blood-derived cell populations including monocytes, dendritic cells, granulocytes, T, B, and natural killer cells, and others [18]. Following the manufacturer’s protocol for permeabilization, the optimal incubation time was found to be 18 min (see Methods). The most closely related tissue evaluated directly by the manufacturer was human heart for which 12 min was reported (10 × Genomics, user guide CG000238 Rev E). Based on the heterogeneity of UMI and gene counts between muscle and mononuclear connective tissue cells, future studies should investigate whether adjusting permeabilization time may increase UMIs in connective tissue-rich spots without negatively affecting transcripts of muscle cells. Furthermore, based on these data, we recommend not to pool samples of unequal section areas during library construction nor sequencing to avoid even more unequally distributed sequencing depths and saturations (Table 1).

While the Visium technology presented as a useful alternative to sc/snRNA-seq because it allowed detection of non-nucleic RNA present in muscle fibers, a limitation was that the transcripts in each capture spot were typically derived from multiple neighboring fibers along with mononuclear cells residing in the adjacent endo- and perimysium. This shortcoming should be considered when analyzing Visium data (Table 1). As a current example, predominant MYH quantification per spot will likely not replace the histological gold standard to quantify muscle fiber types until single fiber resolution is available. To overcome this lack of resolution, previous studies have attempted to determine different cell types per spot and allocate specific transcripts to a specific cell type by combining this spatial technology with sc/snRNA-seq datasets and/or novel deep learning tools [18, 36, 37].

Lastly, the different expression pattern of the regenerative markers MYH8 and MYL4 [34] in healthy vs. torn supraspinatus muscle and even within torn muscle is highly interesting because the degeneration of rotator cuff is still considered irreversible in human [15, 38] and animal models [28, 39], and it is unclear why certain fibers appear to regenerate while others do not. However, the current dataset was intended to evaluate the use of the technology and its nature only allows qualitative analysis. A new dataset including a larger cohort of subjects will be needed for quantitative comparison of healthy vs. torn vs. repaired rotator cuff and analysis of the transcriptional pathways involved in degeneration, regeneration, or lack thereof, in this model. In addition to increased sample numbers per time point, the clinically relevant time point of repair needs to be added along with protein or histological findings to support the transcriptional data.

In summary, these data suggested that Visium may be a valuable technology in the rabbit skeletal muscle translational model as; (1) transcriptional heterogeneity matched the underlying tissue type based on H&E which is routinely used on clinical samples, (2) it can retrospectively be performed in tissue that has been stored for over 6 years, (3) it allows classic skeletal muscle applications, such as assessing myosin heavy chain portions, and (4) it may lead to novel discoveries that go beyond combining standard histology with bulk RNA sequencing or qPCR. Moving forward, larger datasets that allow clinically and pathologically relevant conclusions and more quantitative analyses of different time points and treatments using this technology are needed in skeletal muscle.

## Data Availability

The datasets are publicly available on Gene Expression Omnibus (GEO GSE210773).
